# The Relevance of Ecological Transitions to Intelligence in Marine Mammals

**DOI:** 10.3389/fpsyg.2020.02053

**Published:** 2020-09-08

**Authors:** Gordon B. Bauer, Peter F. Cook, Heidi E. Harley

**Affiliations:** ^1^Division of Social Sciences, New College of Florida, Sarasota, FL, United States; ^2^Mote Marine Laboratory, Sarasota, FL, United States; ^3^The Seas, Epcot^®^, Walt Disney World^®^ Resorts, Lake Buena Vista, FL, United States

**Keywords:** intelligence, marine mammals, cetaceans, pinnipeds, sirenians, brain

## Abstract

Macphail’s comparative approach to intelligence focused on associative processes, an orientation inconsistent with more multifaceted lay and scientific understandings of the term. His ultimate emphasis on associative processes indicated few differences in intelligence among vertebrates. We explore options more attuned to common definitions by considering intelligence in terms of richness of representations of the world, the interconnectivity of those representations, the ability to flexibly change those connections, and knowledge. We focus on marine mammals, represented by the amphibious pinnipeds and the aquatic cetaceans and sirenians, as animals that transitioned from a terrestrial existence to an aquatic one, experiencing major changes in ecological pressures. They adapted with morphological transformations related to streamlining the body, physiological changes in respiration and thermoregulation, and sensory/perceptual changes, including echolocation capabilities and diminished olfaction in many cetaceans, both in-air and underwater visual focus, and enhanced senses of touch in pinnipeds and sirenians. Having a terrestrial foundation on which aquatic capacities were overlaid likely affected their cognitive abilities, especially as a new reliance on sound and touch, and the need to surface to breath changed their interactions with the world. Vocal and behavioral observational learning capabilities in the wild and in laboratory experiments suggest versatility in group coordination. Empirical reports on aspects of intelligent behavior like problem-solving, spatial learning, and concept learning by various species of cetaceans and pinnipeds suggest rich cognitive abilities. The high energy demands of the brain suggest that brain-intelligence relationships might be fruitful areas for study when specific hypotheses are considered, e.g., brain mapping indicates hypertrophy of specific sensory areas in marine mammals. Modern neuroimaging techniques provide ways to study neural connectivity, and the patterns of connections between sensory, motor, and other cortical regions provide a biological framework for exploring how animals represent and flexibly use information in navigating and learning about their environment. At this stage of marine mammal research, it would still be prudent to follow Macphail’s caution that it is premature to make strong comparative statements without more empirical evidence, but an approach that includes learning more about how animals flexibly link information across multiple representations could be a productive way of comparing species by allowing them to use their specific strengths within comparative tasks.

## The Relevance of Ecological Transitions to Intelligence in Marine Mammals

Since the birth of psychology, scientists have debated the power of associationism as the central mechanism behind “the science of mental life” (James, 1890/1952, p. 1). James began his seminal psychological work by contrasting three different possibilities for describing the human mind: soul, associative processes dictated by experience, and the innate and developed structure of the human mind as a framework that constrains how we process information. Macphail (1982, 1987), after a comprehensive review, concluded that an objective assessment of the vertebrate literature indicated that learning only occurred through a limited set of processes, primarily associative (i.e., habituation, classical, and instrumental conditioning). He further indicated that, restricting comparisons to associative processes, there were no differences in intelligence among vertebrates with the exception of humans, and he speculated that difference might be attributable to language, essentially discounting specific cognitive adaptations to distinct niches. Of course, the circumstances in which animals perform and the underlying mechanisms they use are actually the province of psychology, and Macphail’s assertion does not inspire a productive comparative research agenda going forward. Here, we explore a broader framework for interpreting intelligent behavior in animals using widely studied marine mammals (bottlenose dolphins, sea lions, harbor seals, and West Indian manatees) as examples. Their evolutionary history, notably the transition of terrestrial mammals into marine species, forced many adaptations including unique sensory systems, complex social organization, and neurobiological extremes. In addition, many of these animals show flexible cognition, at least, comparable to what has been observed in primates.

Macphail’s definition of intelligence, limited to associative processes, is not consistent with common conceptualizations of human intelligence by experts or the lay public. Expert conceptualizations of human intelligence are multifaceted and include adaptation to the environment, mental processes, and higher order thinking (e.g., reasoning, problem solving, decision-making, and metacognition; review in Sternberg, 2003). Studies of lay ideas of intelligence in the United States identify factors such as speed of processing, practical problem solving, verbal ability, non-verbal reasoning, numerical reasoning, and social competence (Sternberg et al., 1981; Chen and Chen, 1988). Unfortunately, these definitional factors do not hold up well cross-culturally (Nagoshi, 1987), and there is no strong consensus among psychologists on what the components of intelligence should be, illustrating the problem of generalizing from any single human tradition. The origin of the concept of human intelligence and practical applications in individual differences (Binet and Simon, 1916; McNemar, 1964) suggest questionable utility for interspecies comparisons, unless we use individual variability itself, which may be a hallmark of an intelligent species, as a comparative measure. In addition, Mackintosh (1998) notes that associative learning as described by Macphail bears striking similarities to human implicit learning (e.g., Reber, 1993), an area typically not addressed on intelligence tests. This sets a conundrum for comparative psychologists because intelligence defined for humans excludes implicit (associative) processes, and so intelligence would then appear to lie outside the realm of comparative psychology and Darwinian evolution.

Comparative researchers responding to Macphail’s null hypothesis of no species differences in intelligence emphasized that intelligence consists of multiple facets including sensory and perceptual processes, memory, spatial relations, concept formation, rule learning, and tool use (Goldman-Rakic and Preuss 1987; Hodos, 1987; Shettleworth, 1987; Rilling, 1990; Walker, 1990). Bullock (1986) suggested that candidates for investigation might include flexibility in interacting with the environment, social interactions, communication, and difficult, higher forms of cognition, plus problem solving across all the categories. In addition, he considered acquired knowledge essential to considerations of intelligence. Goldman-Rakic and Preuss (1987) and Vauclair (1990) among others also suggested that representation rather than association might be a more productive approach. Representations, based on the environmental information that animals extract through their sensory-motor systems and then organize perceptually and cognitively, vary widely across species and facilitate intelligent behavior. Associations between representations and the breadth and flexibility of those representations may be especially relevant for marine mammals, who become interesting due to their operating so effectively in two vastly different perceptual media – water and air – where they must recruit sensory-motor systems developed differentially for this split life. More recent approaches to animal intelligence retain a multifaceted approach (e.g., Roth and Dicke, 2017).

In considering the intelligence of marine mammals, we start with the assumption that marine mammals have the basic associative processes identified by Macphail (1982, 1987) and demonstrated ubiquitously in marine mammal training (Pepper and Defran, 1975), entertainments at commercial oceanaria, and numerous studies. We take an evolutionary stance that the transition from a terrestrial to an aquatic environment modified sensory and perceptual processes, as well as the flexibility and processing speed of other cognitive processes contributing to intelligence. We also investigate the implications of marine mammal neurobiology in the manifestation of intelligent behavior. For us, intelligence is the effectiveness by which one deploys cognitive processes including sensation and perception, instantiated in the central and peripheral nervous systems, and studied through investigations of behavior.

## Marine Mammals

Marine mammals can be characterized as the mammals that depend primarily on the marine environment for survival (Rice, 1998). This list could include marine otters (*Lontra felina*), polar bears (*Ursus maritimus*), Arctic foxes (*Vulpes lagopus*), and fishing bats (*Noctilio leporinus*), which feed on marine prey, but much more is known about the senses and cognition of cetaceans (whales and dolphins), pinnipeds (seals, sea lions, and walruses), and sirenians (manatees and dugongs), so our focus will be on these orders. There are species within these orders that are exclusively freshwater such as the river dolphins, family Iniidae and Platinistidae, and some of the manatee species such as Amazonian manatees (*Trichechus inunguis*), but by and large these orders are marine. Within each order only a few species have been studied, so some caution needs to be observed in generalizing across species, but these few species serve to provide a working base with which to compare other species. In addition, sample sizes for laboratory experimentation are small, frequently only one or two subjects, so conclusions are likely to be modified as more subjects are studied.

The ancestors of cetaceans (whales and dolphins) and sirenians (sea cows) made a major transition from a terrestrial to an aquatic environment during the Eocene (~50 million years ago). The pinnipeds (seals, sea lions, and walruses) made a partial transition more recently during the late Oligocene (~26–23 million years ago) and remain amphibious, feeding at sea but reproducing on land. These three orders, which constitute the most studied groups of marine mammals, responded to new ecological pressures with numerous adaptive changes in morphology, physiology, behavior, and sensory/perceptual processes, thereby shifting the information they could gain about the world and thus their representations of it.

Morphological changes included streamlining the body to reduce drag, including loss or reduction in hind limbs and modification of forelimbs, various other skeletal modifications, loss, reduction or modification in pelage, and internalization of male reproductive organs. Respiratory mechanisms had to meet simultaneous demands for the combination of in-air breathing with diving, often to great depths under great physical pressure. Circulatory systems were modified to maintain warm body temperature in cold water environments. Many species developed group social structures and cooperative systems for foraging and defense in an environment with few places to hide either for purposes of prey ambush or predator avoidance, especially for animals coupled to the surface for respiration. Communication systems emphasized auditory and tactile channels, while de-emphasizing or modifying visual systems, which were limited by low light and turbidity underwater and sharp transitions in brightness at the surface. Olfactory systems that evolved on land had limited utility underwater. Novel sensory processes, such as echolocation and exquisite senses of touch, were shaped by natural selection to facilitate foraging and orientation in a marine environment. An evolutionary perspective suggests that adaptation to the aquatic realm overlain on a terrestrial foundation likely affected an array of behavioral and cognitive intellectual processes, preserving some attributes while modifying others.

Bullock (1986) provides an entry to a comparative assessment of intelligence beyond associationism by presenting a broad palette of candidate domains for the investigation of animal intelligence. We have selected from that palette to emphasize flexibility in problem solving, the neural plasticity that underlies flexibility, and knowledge. Curiously, Macphail (1987) also emphasized the generality of human intelligence, as well as its dependence on experience. Knowledge is a little studied topic in marine mammal science, but we can identify mechanisms that would allow the accumulation of knowledge: the resolution (perceptual detail) of sensory systems, the speed of information transfer by imitation, retention over long time periods, and facility at problem solving. We have organized this information into four categories: sensation and perception, social learning, flexibility of cognitive processes, and the brain.

## Sensation and Perception

Sensory-motor experiences provide a foundation for intelligent thought by providing insight to the quality, range, and resolution of animal worlds or *Umwelten* (von Uexküll, 1934/1957), the detail creating the representations operated on during cognitive processing to produce intelligent behavior. Early researchers (Galton, 1883; Cattell, 1890; Spearman, 1904) considered sensory discrimination as integral to human intelligence, but their view failed to gain traction in mental measurement (Deary, 1994; Sternberg, 2003). Nevertheless, subsequent research provided support for this sensory hypothesis: for example, Deary et al. (2004) reported a high correlation between general sensory discrimination (representing shared variance across several modalities) and fluid intelligence (Cattell, 1963), which is closely related to working memory (Kyllonen and Christal, 1990; Salthouse and Pink, 2008). Intelligence differences between animal species may even more strongly reflect sensory processing, since sensory differences between species are more likely to be greater than differences within a single species, such as humans.

The marine environment places specific demands on sensory perception. The slow rate of diffusion of chemical compounds in water limits their utility to marine mammals compared to terrestrial mammals living where olfactants are rapidly dispersed. The olfactory systems of terrestrial mammals, designed to detect and discriminate airborne compounds, are of reduced importance to animals spending substantial time underwater. Taste may be relevant but the overall sense of flavor (i.e., combined effect of taste and smell) is probably lessened to the extent that olfaction is unavailable. Underwater vision is constrained by the limits of photic transmission in water, and it loses much of its relevance at depth or in turbid environments where light is limited. Touch provides advantages underwater for sensing hydrodynamic movement caused by currents or distortions in water flowing past objects, as well as for close contact investigation of items. Sound in water travels close to 4.5 times as fast as sound in air and can be conveyed with fidelity over great distances. The long wavelengths of lower frequency sounds allow them to pass around objects that block light transmission, and high frequency sounds are capable of transmitting detailed information over shorter distances. Adaptations for enhanced acoustic and tactile processing required for life underwater not only fostered new sensory mechanisms for gaining important information but also pushed speed and range of processing to new heights due to the physics of sound transmission and pressure changes in water.

### Cetaceans

There are over 80 species of odontocetes (toothed whales) living in diverse environments. The river dolphins, who live in muddy waters thick with particulate matter, have extremely poor eyesight. For example, Platanistidae, the Southeast Asian river dolphins, are probably capable of seeing only degrees of brightness and the *Inia*, the South American river dolphins, have visual acuity of over 40 arc min (Mass and Supin, 1989). Bottlenose dolphins (*Tursiops truncatus*) have considerably better resolution, useful in the frequently more transparent water of coastal regions. Underwater visual acuity for bottlenose dolphins is about 8.5 min and in-air is 12.5 min (Herman et al., 1975). This reasonably good acuity underwater and in-air is surprising because of the differential role the cornea plays in refraction underwater (practically none) and in air where it is the primary refractive component of the eye. An eye adapted for vision underwater should not be able to focus in air and vice-versa, without specific adaptive mechanisms, which dolphins have (Herman et al., 1975). Dolphins, like other marine mammals tested, are monochromats (Ahnelt and Kolb, 2000) who see the world in shades of gray (Madsen and Herman, 1980), although there is evidence that they may have some color perception, presumably mediated by the differential sensitivity of rods and the single cone-type (Griebel and Schmid, 2002). Dolphins depend on vision to build their representations of the world, their *Umwelten*. For example, they integrate information from multiple sensory systems, like vision and echolocation, to represent objects (e.g., Harley et al., 1996), and they can discriminate among photographs and video of fish underwater using vision alone, likely an important ability for stealthy foraging (Harley et al., 2019).

Dolphin hearing is exceptional (reviews in Supin et al., 2001; Au and Hastings, 2008) ranging from 0.15 to 200 kHz, an upper limit over three octaves higher than that of humans. They are excellent at sound localization with 0.5–4 degrees of resolution. They have a temporal processing rate, the ability of the nervous system to map sound intervals, as measured by auditory brainstem responses, over 1,500 Hz for amplitude modulated sounds (compared to a rate of 50 Hz for humans). These evoked potential measures provide only indirect measures of temporal processing. Behavioral tests, direct tests of the ability of dolphins to discriminate sound intervals, indicate that they have a temporal integration time of an order of magnitude less than humans do (Supin et al., 2001). Dolphins are also active echolocators that have the ability to make subtle distinctions among object characteristics, e.g., they can discriminate cylinders that vary in wall thickness by less than a millimeter (Au and Pawloski, 1992). They can also recognize an equivalence between their visual and echoic experiences of objects (Harley et al., 2003) and share echoic information with nearby eavesdropping dolphins about object identity (Xitco and Roitblat, 1996). Clearly, their representations of objects are fine-tuned and flexible.

The sense of touch in dolphins has not been investigated to the same extent as in other marine mammals but electrophysiological measures of skin response show greatest sensitivity around the head (Ridgway and Carder, 1990, 1993) with sensitivity comparable to human lips and fingers, sufficient to detect underwater turbulence (Kolchin and Belkovich, 1973). Hair, important for touch in other marine mammals, has not been investigated well in cetaceans, probably because of its infrequent appearance among odontocetes, where it is found only on the rostrum of river dolphins and some neonates of other species. Sensory hairs are found on the rostrums of baleen whales, but they are difficult to study in these large, pelagic animals, although the structure of hairs of right whales appear to be adapted for detection of small prey such as plankton (Murphy et al., 2015).

Cetaceans have missing or greatly reduced olfactory bulbs and ethmoturbinates. Their taste buds are few. Nevertheless, they have low thresholds for sour (citric acid) and bitter (Nachtigall and Hall, 1984; Friedl et al., 1990; Kuznetsov, 1990). They also can detect salt.

### Pinnipeds

Although a large number of species comprise the pinnipeds (seals, sea lions, and walruses), most sensory research has been conducted on the California sea lion (*Zalophus californianis*) and harbor seal (*Phoca vitulina*). The vision of the pinnipeds may be most notable for relatively high acuity both in air and underwater. The large, curved orbit of the lens focuses light on the retina underwater. This would lead to myopic (near-sighted) vision in air, except the cornea in pinnipeds contains a flattened area over the pupil reducing or eliminating corneal refraction in air (West et al., 1991; Miller et al., 2010). The underwater and in-air acuity of the sea lion are equivalent at moderate and high brightness at 4.7–7 arc min, but underwater vision is better under dim light conditions. Seal vision is similar at 5–8 min. Pinnipeds are monochromats, and therefore, do not have dichromatic color vision as do most terrestrial mammals (Ahnelt and Kolb, 2000), although a weak form of mesopic color vision in seals has also been reported (Oppermann et al., 2016). These reports of a weak form of color vision based on rod-cone spectral sensitivity differences (Griebel and Peichel, 2003) have been challenged (Scholtyssek et al., 2015).

Audiograms for pinnipeds tend to have considerable variability among studies, perhaps attributable to small sample sizes (frequently just one animal) and individual differences, but in general, the frequency range for harbor seals is about 0.2–72 kHz (Kastelein et al., 2009), with sea lions having a somewhat lower upper limit. Early reports of hearing by pinnipeds suggested that underwater hearing was superior, but more recent evidence suggests that they are similar with both having low threshold levels (Reichmuth et al., 2013), a more understandable relationship given pinnipeds’ amphibious existence and terrestrial ancestry. Pinnipeds demonstrate sensitive mechanoreception both in the active (haptic) and passive modes, which they use for detecting hydrodynamic stimuli. They can discriminate size and shape by active touch (Dehnhardt, 1994; Dehnhardt and Dücker, 1996) and detect water movement at detection thresholds under a micron of particle displacement (Dehnhardt et al., 1998; Dehnhardt and Mauck, 2008). Their high sensitivity to hydrodynamic stimuli allows both seals and sea lions to track fish by the turbulence they generate in swimming. While sea lion vibrissae appear to be more sensitive than those of phocids to relatively low frequency vibrations in the water, harbor seals have shown greater ability at following complex wakes over longer periods of time (Gläser et al., 2011), perhaps due to differences in vibrissal structure (Hanke et al., 2010; Witte, 2012).

The olfactory bulbs of pinnipeds are reduced in size, and there are fewer nasal turbinates. Nonetheless, scent recognition is a demonstrated feature of individual recognition in pinnipeds, particularly well-studied in mother-pup identification, and likely relevant for reproductive behavior in some species (Pitcher et al., 2011). Gustation has hardly been studied. There are taste buds on the tongue, albeit fewer than among terrestrial mammals. Despite the unimpressive anatomy associated with the chemical senses, sea lions detect sour, bitter, and salt (Friedl et al., 1990). They also have low discrimination thresholds for saline solutions (Sticken and Dehnhardt, 2000).

### Sirenians

West Indian manatees have modest visual acuity of approximately 20 arc min (Mass et al., 1997; Bauer et al., 2003) and probably limited visual tracking capabilities (Samuelson et al., 2012). Unlike many of the cetaceans and pinnipeds studied, they lack a tapetum lucidum for enhancing light sensitivity, but also unlike them have dichromatic color vision (Griebel and Schmid, 1996; Ahnelt and Kolb, 2000; Newman and Robinson, 2006). Preliminary evidence from streak retinoscopy indicates emmetropic to hyperopic vision both underwater and in-air (Samuelson et al., 2012). They lack a vomeronasal organ and their neurophysiology suggests modest olfaction (review in Reep and Bonde, 2006). They have a higher density of taste receptors than dolphins (Yamasaki et al., 1980), but the psychophysics of taste and other chemical senses has not been investigated. Auditory capabilities include about an eight-octave frequency range extending from about 0.25 kHz into the ultrasonic range over 70 kHz (Gaspard et al., 2012), a high temporal processing rate (Mann et al., 2005), and good sound localization (Colbert-Luke et al., 2015). Manatees are the only mammal known to have exclusively sensory hairs (vibrissae) covering their entire body. Manatees’ sense of touch is highly sensitive with Weber fractions between 0.025 and 0.14 (Bachteler and Dehnhardt, 1999; Bauer et al., 2012). At low frequencies, they can detect hydrodynamic particle movement under a micron with an order of magnitude greater sensitivity rostrally (Gaspard et al., 2013, 2017).

Although formal, behavioral experiments have not been done, the sensitivity and resolution of the manatee senses of hearing and touch suggest the ability to discriminate fine detail, which might allow for orientation by auditory and tactile scene analysis. Masking experiments reveal enhanced hearing in noise as indicated by low critical ratios, especially within the range of the second and third harmonic (Gaspard et al., 2012), which in conjunction with field studies identifying signature vocalizations, suggest that manatees might acoustically differentiate among individuals (Sousa-Lima et al., 2002). Although the physiology of chemoreception is unimpressive, naturalistic observations of tracking estrus females and locating fresh water in a saltwater milieu suggest that chemical senses might be more prominent than expected.

In summary, the three orders of marine mammals display visual modifications appropriate for maintaining an adaptive level of visual acuity in underwater and in-air environments. Their sense of hearing allows detailed temporal perception, exquisite in the case of echolocating cetaceans. The active touch sense facilitates fine textural discrimination in pinnipeds and manatees. In the passive touch mode, harbor seals and sea lions can follow the trail of residual turbulence left by swimming fish. The sensitive mechanosensory systems of manatees and dolphins are likely to be similarly sensitive to water movement. The chemical senses remain to be explored more thoroughly.

One way to think about many of these sensory characteristics (e.g., high frequency hearing) are as adaptations for particular niches. Byrne (1995, p. 34), however, argued that viewing adaptations as intelligence adds nothing explanatory, so suggests that “intelligence” be reserved for something more restricted, a “… quality of flexibility that allows individuals to find their own solutions to problems.” We agree that sensory adaptations by themselves are not intelligence, but when integrated with systems that connect senses to motor responses (c.f., von Uexküll, 1934/1957), and when these connections generate complex behavior, intelligence emerges. Furthermore, sensory systems that are multimodal can be linked by common representations, which might provide a useful avenue for considering intelligence. For example, a pit viper that uses heat as a single indicator of prey or predator and strikes at it, whether it is a mouse or a warm water-filled balloon, has a narrow perceptual world. In contrast, a cat might integrate its good visual resolution, keen sense of smell, and high frequency hearing to represent the warm object as a mouse. An *Umwelt* built at this level of complexity provides more tools for problem solving and adaptability – more opportunities to build a better mousetrap.

The marine environment promoted the development of high resolution auditory and tactile senses in marine mammals, and in the case of the former, it fostered high speed processing. These adaptations, in conjunction with good visual acuity found in many, but not all, species, facilitated a general sensory foundation for multimodal, rapid, integrated information processing. Furthermore, the selective pressures of an aquatic environment to develop general sensory systems suggest the possibility of generating richer representations and perhaps something akin to the fluid intelligence capacity described in humans (Deary et al., 2004). Fluid intelligence, per se, has not been assessed to our knowledge in marine mammals, but its correlate, working memory, has been well-investigated (e.g., Thompson and Herman, 1977, 1981).

## Social Learning

Dim light and the efficiency of sound transmission in the underwater environment favor acoustic (and possibly tactile) communication among marine mammals. The structural characteristics of vocalizations by marine mammals are well described in the literature, but it is only among the cetaceans that we find substantial investigation of the cognitive aspects of communication, especially vocal mimicry. Dolphins also demonstrate flexible behavioral mimicry which may be unique among non-human mammals in the variety and flexibility of both vocal and behavioral copying, although these capabilities have been demonstrated to some extent in an African gray parrot (Moore, 1992), as well. Little is known of the cognitive aspects of pinniped vocalizations, although a single case study of a harbor seal that spoke several phrases in English (Ralls et al., 1985; Deacon, 1997) suggests that it is an area worth greater attention. The ability to engage in social learning not only expands avenues for gaining new information and skills, but also pushes individuals to decode the actions of conspecifics, a rich area for cognitive growth. When social learning occurs through mimicry, this decoding requires a representation of a social partner that applies in a fine-tuned way to oneself. Although some behavioral copying can be learned slowly through trial and error, consistent with Macphail’s perspective, laboratory evidence of rapid acquisition, including single trial learning, suggests more efficient mechanisms.

### Cetaceans

Herman (1980, 2002) and Whitehead and Rendell (2015) provide several, thorough reviews of both vocal and behavioral mimicry. Therefore, in this section we will provide brief summaries of research previously reviewed and updates of more recent literature.

#### Vocal

Marine mammals show remarkable flexibility in vocal copying, e.g., with human-like spontaneous vocalizations in beluga whales (*Delphinapterous leucas*; Ridgway et al., 2012) and dolphin-like vocalizations by killer whales (*Orcinus orca*) who had dolphin pool-mates (Musser et al., 2014). Wild social groups of killer whales share call types (Ford, 1991). Young dolphins born in human environments incorporate trainer’s whistles into their repertoires (Miksis et al., 2002). Dolphins also spontaneously mimic computer-generated sounds (Herman, 1980; Richards et al., 1984; Richards, 1986; Reiss and McCowan, 1993), both narrow and broadband. Dolphins naturally copy each other’s identifying whistles, individually distinctive signature whistles that serve as contact signals (Caldwell and Caldwell, 1965; Tyack, 1986; Caldwell et al., 1990). These whistles are learned, unique identifiers discriminable by other dolphins (Harley, 2008), and used on meeting in the wild (Janik et al., 2006). Dolphins can vocally mimic on command in controlled laboratory settings, including the sound bursts of human speech (Lilly, 1965; Lilly et al., 1968), and sine waves, similar to natural sounds, but also atypical sounds like triangular and square wave tonal patterns, sometimes going beyond copying to mimic amplitude modulations and transients at the onset of tonal stimuli, as well as transposing sounds by an octave (Richards, et al., 1984).

Vocalizations are not the only behaviors showing evidence of dolphin mimicry and perhaps other forms of social learning. Synchrony in swimming, respiration, and leaping is a common feature of wild dolphin behavior (Connor et al., 1992a, 2006b). Synchrony occurs immediately after birth (Cockcroft and Ross, 1990) as dolphin calves swim continuously (Lyamin et al., 2005; Sekiguchi et al., 2006) in the slipstreams of their mothers. Calf synchronous swimming with other dolphins in the social group appears later in development (Fellner et al., 2012). The early development of synchrony may support social learning capabilities (Whiten, 2001; Fellner et al., 2006; Hastie et al., 2006; Whitehead and Rendell, 2015) and act as a means of social affiliation (Connor et al., 2006a,b; Perelberg and Schuster, 2009) and cultural transmission of information (Bauer and Harley, 2001; Whiten, 2001; Fellner et al., 2012).

There is a rich anecdotal literature on cetacean behavioral imitation, for example, captive bottlenose dolphins (*Tursiops aduncus*) copying the sleeping posture of a Cape fur seal (*Arctocephalus pussilus*); recruiting feathers, expelling bubbles and making scuba noises to mimic human divers cleaning; and acquiring and releasing a mouthful of milk, like a smoke cloud, at smokers standing by a pool window (Tayler and Saayman, 1973). In commercial shows, a false killer whale (*Pseudorca crassidens*) learned the routines of a pilot whale and two rough-toothed dolphins by observation (no training involved; Brown et al., 1966), and a bottlenose dolphin copied the unique, spiraling leap of a spinner dolphin (*Stenella longirostris*) introduced to its tank, atypical for a bottlenose. Another example suggesting emulation of a routine occurred at Kewalo Basin Marine Mammal Laboratory (Bauer, personal observations, 1979–1980). The routine for training the dolphins for object “names” and actions included a tonal secondary reinforcer for correct behaviors and then fish reinforcement. During the sessions, the dolphins would drop fish to the tank bottom, and occasionally, for incorrect trials, the dolphin itself would whistle the secondary reinforcer and eat a stockpiled fish.

Ostensibly insightful or otherwise intelligent behavior frequently attracts human attention, despite absence of knowledge of how these behaviors developed. Often, trial and error mechanisms explain the behaviors (Macphail, 1982; Shettleworth, 2010). Here, controlled experimental studies support the anecdotal evidence highlighting the flexibility of dolphin cognition. Young bottlenose dolphins in a “Do this…” paradigm mimicked humans modeling a diverse array of behaviors, some on the first trial (Xitco, 1988; Herman, 2002), even with dramatic differences in morphology (e.g., legs vs. fluke and flipper vs. arms), which present a concordance problem. Xitco et al. (1998) later brought imitation under control of a hand signal designating mimic in a study of dolphin-dolphin imitation with two dolphins. Importantly, the model was given hand signals to do other behaviors in addition to mimic, so that mimicry was clearly under stimulus control of an arbitrary signal. The experimental design included training on a set of behaviors and testing on a set of different, untrained but familiar behaviors, and finally on a set of novel behaviors. Both untrained familiar behaviors and novel behaviors were copied, some on the first trial. Xitco also demonstrated that the dolphins could successfully copy behaviors after delays up to 80 s. The mimicry of novel behaviors met Thorpe (1963) criterion for imitation: learning a new behavior by copying. Bauer and Johnson (1994) partially replicated this study, although without demonstrating mimicry of novel behaviors. Major differences in subject experience could have easily accounted for this discrepancy.

Later, Herman et al. demonstrated that dolphins could copy a human model standing in air and a previously performed behavior (self-mimicry; Mercado et al., 1998). The experiments indicated that dolphins were responding to visual cues but left open the possibility that the dolphins might, in addition, respond to auditory and tactile (water flow) cues. Jaakola et al. (2010, 2013) demonstrated that dolphins could perform modeled behaviors even when they were wearing eyecups blocking vision, using passive listening with dolphin models and echolocating human models.

In summary, dolphins exhibit robust mimetic abilities, both vocally and behaviorally, an apparently unique combination among non-human mammals. They copy sounds of conspecifics, computer generated sounds, and qualities of human speech. They copy a rich variety of behaviors modeled by different species with different morphologies. They mimic models in water and out of water. They mimic spontaneously and under stimulus control. Their mimicry is exhibited to visual stimuli alone and to acoustic and possibly tactile stimuli. They mimic synchronously and after delay, demonstrating the persistence of the representation. All of these factors argue that dolphins have a conceptual understanding of imitation. Herman (2002, p. 100), in a review of dolphin imitation, asks:

What does it mean to have a generalized concept of “imitate”? It implies that the capacity is not reserved or restricted to functionally significant events, or to events tied to the organism’s natural repertoire, ecology, or habitat, but is broadly understood as applicable to any arbitrary experienced event. The dolphin is obviously an imitative generalist…

Ascertaining how copying behavior functions in the wild is difficult because of the problem of controlling alternative explanations of behavioral acquisition. For example, copying behaviors might reflect contagion, social facilitation, stimulus or response enhancement, observational conditioning, or matched-dependent behavior, which are expressions of already existing behaviors or behaviors easily explained by trial and error learning. These are difficult to discriminate from true imitation that requires actually learning new behaviors (reviews in Whiten and Ham, 1992; Zentall, 1996). Nevertheless, we can propose promising candidates for social learning in all its forms by looking at wild behavior.

Wild marine mammals are highly flexible foragers. Foraging techniques found in limited groups of the same species present interesting examples of cooperation that suggest social learning. For example, symbiotic fishing between humans and dolphins was reported by Pliny the Elder (~70 AD) and more recently in Australia, Brazil, Myanmar, India, and Mauritania (review in Whitehead and Rendell, 2015). Typically, dolphins herd fish toward fishermen who capture them in nets and wild dolphins capture the fish concentrated between them and humans. The origins of these cooperative fishing ventures are unknown, but the outcome appears to be beneficial for both species. There is also some evidence that California sea lions use dolphins to locate large schools of fish for predation (Bearzi, 2006). Another example is provided by small groups of sponge feeding dolphins (*T. aduncus*) in Shark Bay Western Australia, where these dolphins carry sponges on their rostrums, presumably as protection from fish spikes on the murky bottom (Mann et al., 2012). At least two unrelated subgroups share the behavior, suggesting some social learning, although there is some familial relatedness within each subgroup. These candidates for acquisition of knowledge through social learning might be explained by vertical transmission, parent to offspring. A case broadening the sources of knowledge within a group has recently been provided in a study of the unusual behavior of “shelling,” also by bottlenose dolphins (*T. aduncus*) in Shark Bay. In “shelling” a dolphin drives fish into large shells, takes the shell to the surface, and then shakes the fish out into its mouth. Integrating behavioral, genetic, and environmental data, Wild et al. (2020) demonstrated that the behavior is transmitted horizontally (i.e., relationships other than parent-offspring). Both vertical and horizontal transmission of foraging behavior enhances the dispersion of knowledge and increases flexible responding.

Killer whales are apex predators feeding on a wide variety of prey (e.g., beaked whales, salmon, herring, seals, cephalopods, gentoo and chinstrap penguins, humpback whales, gray whales, gray seals, blue whales, sea turtles, minke whales, emperor penguins, elephant seals, sharks, deer, and moose). Different prey require different hunting techniques including corralling, swimming onto beaches, and collaboratively creating waves to wash prey off ice floes (Visser et al., 2008). In another social sphere, male dolphins synchronize and coordinate both vocal (Moore et al., 2020) and motor behavior to control and protect access to females (Connor et al., 2006b).

Although we do not have controlled, laboratory experimental data on baleen whale behavior, in the wild they engage in a variety of cooperative behaviors such as synchronous swimming, cooperative foraging, and memory for migratory destinations that suggest the possibility of social learning, but in most cases, instinctual responding cannot be ruled out. A notable exception is evidence that humpback whale song (*Megaptera novaeangliae*) is learned socially (for reviews of humpback whale song, see Payne, 1983; Whitehead and Rendell, 2015). Humpback males sing at the tropical/subtropical termini of their annual migrations from polar regions. Elevated testosterone and increased mating behavior in these regions suggest that the songs have reproductive functions. The songs range over seven octaves (~30 Hz to 4 kHz) and have units, phrases, and themes organized in a hierarchical structure. The units include tonal whistles and broadband sounds lasting from 0.15 to 8 s. Generally, there are fewer than 10 themes in a song, but for any one song, the order and number of themes are fixed, although the number of phrases may vary. The song, which may last from 10 to 30 min, is repeated, continuing for many hours.

Social learning of songs is indicated by several factors. Humpback whale populations are discrete, with relatively little exchange with other populations. The songs are the same for all members of a population and they change over the course of a season and between seasons (i.e., annually) by dropping or adding themes. Furthermore, evidence from the South Pacific indicates that songs are transmitted east to west, while at the same time, there is little east to west movement by individual whales. Therefore, the movement of song represents a transfer of information, not movement of individuals (Garland et al., 2011).

Learning, remembering, and producing these complex, changing songs suggest substantial cognitive demands on male whales. Interestingly, Guinee and Payne (1988) reported that they had found multi-themed sub-phrases that formed similar beginnings and endings of adjacent phrases, a phenomenon they characterized as rhyme-like. These rhyme-like patterns were positively correlated with the number of themes (i.e., the amount of material to be remembered) but not duration, suggesting a mnemonic function like that found in human recitation of long, complex works.

### Pinnipeds

Less is known regarding vocal learning in pinnipeds than in cetaceans, and there have been very few experiments probing behavioral motor imitation. However, there are emerging observations that suggest that pinniped species may demonstrate a rich range of vocal learning capabilities.

As reviewed by Reichmuth and Casey (2014), there is growing evidence – predominantly gathered from observational field studies – for vocal learning in phocid pinnipeds. This includes regional variability in vocalizations of Weddell seals, leopard seals, harbor seals, harp seals, and bearded seals and raises the possibility of social learning influencing development and production of vocalizations in the wild. Implementing the types of developmental cross-fostering studies that have illuminated vocal learning in birds using pinnipeds is logistically and ethically difficult. However, opportunistic observations of a female Northern elephant seal raised in social isolation suggest species atypical call-types, as has been observed in songbirds reared in similar situations.

The famous but singular case of Hoover the harbor seal (Ralls et al., 1985; Deacon, 1997) continues to stoke interest in vocal imitation and flexible learning in phocids. Hoover was orphaned as a pup and rescued by a fisherman, who raised him until he became too difficult to maintain. Hoover was then transferred to the New England Aquarium where he surprised staff and visitors by speaking English phrases, which included, “Hey!, Hey!, Hey!, Hey!”; “Hoova!” (Hoover with a New England accent); “Hey!, Hey!, Get outa there!,” “Hello there,” and “Come over here”; and some speech-like, but indecipherable sounds. It is not clear where and how he learned to “speak.” Based on Hoover’s accent and other factors, Deacon (1997) has suggested that he had learned speech from the fisherman. Since the origin of Hoover’s speech is unknown, we cannot determine if it was copied or learned by trial and error. Hoover is apparently unique among pinnipeds regarding the quality and specificity of his mimicry. What is clear is that for now Hoover is unique among pinnipeds in his mimicry of human speech. Laboratory research probing vocal ontogeny in phocid pinnipeds is ongoing (Ravignani, 2019; Ravignani et al., 2019).

Walruses, which are a separate clade from the phocid (true seal) and otariid (sea lion) pinnipeds, have also been suggested as potential vocal imitators, although more data are needed. In the wild, adult male walruses have been shown to alter song types substantially over subsequent breeding seasons, much as humpback whales do (Sjare et al., 2003).

There are scant data on behavioral motor imitation in pinnipeds, but there is reason to further explore their capabilities. While phocid pinnipeds are typically weaned very rapidly (within a month of birth) and do not have extensive social interactions during development, most of the otariid pinnipeds spend far longer with their mothers prior to weaning, up to 2 years for Steller sea lions (Trites et al., 2006). Walrus pups may spend even longer with their mothers, up to 5 years in some cases (Fay, 1982). The young of most otariid species live in large, crowded, hyper-social rookeries, where they spend much of their day engaging in play behavior with other young. Play is a rich context for social learning, and, indeed, there is some evidence of social learning during Steller pup play (Gentry, 1974). This extended weaning period, during which otariid young achieve significant mastery of open ocean swimming well before they need to hunt on their own, may also allow a period of social observational learning related to hunting behavior of adults. Fur seal pups have been observed overlapping with hunting adults months before they begin hunting on their own (Lowther and Goldsworthy, 2012). The apparent vocal flexibility of phocid pinnipeds, and the extended juvenile period and active play of otariid and odobenid pinnipeds, provide reason to further probe social learning and imitation in pinniped species.

The apparent profusion of social learning and mimicry across cetaceans and pinnipeds is noteworthy, given the frequent difficulty of proving these abilities in laboratory studies with terrestrial mammals. Social learning among sirenians has not been reported to our knowledge. The ecology of marine mammals has generally favored long lives and large group size, both of which may privilege accumulation of social learning across the lifespan. Though each instance of such apparent learning must be investigated, cognitive flexibility is broadly believed to support such rapid and variable learning.

## Flexible Cognitive Processing

Although some cognitive abilities found in marine mammals were modified to adapt to the aquatic environment, many useful attributes were no doubt conserved in the transition from a terrestrial environment. Cetaceans display flexibility across a broad array of learning, memory, and problem solving tasks (reviews in Herman, 1980, 1986; Marino et al., 2007; Mercado and DeLong, 2010; Pack, 2015; Harley and Bauer, 2017), as do pinnipeds (reviewed in Schusterman et al., 2002; Cook et al., 2020).

### Cetaceans

Many odontocete species, e.g., *T. truncatus*, *Delphinus delphis*, *Phocoena phocoena*, *Inia geoffrensis*, and *Lagenorhynchus obliquidens*, display basic discrimination learning abilities, frequently exhibited in studies of sensory detection discrimination thresholds, particularly perceptual systems in the auditory and visual domains (reviews in Nachtigall, 1980, and Au, 1993, of echolocation discrimination learning). Cetaceans have been tested broadly on other cognitive tasks showing that they are proficient at abstract rule learning. Dolphins demonstrated facility in auditory learning sets using hundreds of novel pairs and in reversals of the same pairs (Herman and Arbeit, 1973; reviews in Herman, 1980, 1986; Herman et al., 1993). Both procedures require learning a win stay/lose shift rule. Early efforts with training visual stimuli were not successful (Herman et al., 1969) suggesting a bias toward audition, a possible adaptation to an aquatic environment, but later work suggested that dolphins were capable in both domains. For example, dolphins tested with auditory (echolocated) and visual 2d and 3d planar stimuli successfully solved same/different discrimination problems (Mercado et al., 2000). They demonstrated generalization of the concept by correctly classifying pairs of novel targets in air on the first trial and then transferring this ability to unfamiliar targets presented underwater. They also transferred the same/difference concept from pairs of objects to objects presented in groups of three, in which “same” was represented by three identical objects and different by two identical objects and one different object.

Retention of information by cetaceans has been tested using a variety of short-term and long-term memory procedures. Many of the memory findings are broadly found among species, but it is nevertheless important to establish similarities in intelligent behavior, as well as differences. Dolphins do well on tests of short-term working memory, typically assessed in matching tasks in which a sample stimulus is presented followed by a recognition test in which two or more stimuli, one of which matches the sample, are presented after a delay. Dolphins in artificial “language” testing also performed what were essentially conditional matching-to-sample problems (Herman et al., 1984), in which the sample stimulus was symbolically represented in the test as a sound or hand-sign (A to A') that was paired with an object choice presented among object alternatives (A', B', C', etc.). Dolphins also showed a recency effect for lists of sounds (Thompson and Herman, 1977) and good memory for relative spatial positions (Herman et al., 1984). Long-term memory has not been well-studied. In an investigation of captive dolphins using a habituation-dishabituation design, subjects apparently remembered signature whistles over a period of 15 years (Bruck, 2013). Memory for signature whistles would be an adaptive characteristic for long-lived dolphins living in fission-fusion societies in the wild.

In a creative use of memory, dolphins can acquire an “innovate” rule by correctly doing a self-selected new behavior when signaled to do so. Pryor et al. (1969) reported an early instance of this rule with rough-toothed dolphins (*Steno bredanensis*). On command, the subjects were reinforced for executing a behavior not previously done in the innovation training sessions. The experiment was terminated after 16 reinforced innovative behaviors, when trainers found it hard to discriminate between novel and familiar behaviors. In similar studies, trainers brought novel or not recently performed behaviors under stimulus control (reviews in Kuczaj and Yeater, 2006; Mercado and DeLong, 2010). Difficulty in ascertaining novel behaviors in long-term projects may make “rare behaviors” a better term. Under any circumstances, the “innovate” procedures required learning the opposite of the learning set rule; a win shift strategy is required.

More evidence of dolphins’ flexible cognitive powers occurred at the Kewalo Basin Marine Mammal Laboratory where two dolphins learned an artificial “language,” in which experimenter-created “words” were presented to the dolphins by hand signals or arbitrary computer-generated sounds. The dolphins successfully learned signals representing objects, actions, and modifiers. They also learned that the order of “words” in language-like sequences could indicate different actions. For example, the sequence “hoop pipe fetch,” meaning take the pipe to the hoop, required a different action than “pipe hoop fetch” meaning take the hoop to the pipe. Five-word sentences were created by adding relational modifiers (e.g., surface vs. bottom and right vs. left). Evidence that the dolphins learned a specific grammar, not simply memorizing specific sequences, was indicated by the fact that they could correctly follow behavioral instructions when novel terms were introduced. There is some disagreement concerning whether these dolphins actually displayed language-like learning with syntax (Herman, 1988, 1989) or an associative process (Schusterman and Gisiner, 1988). In either case, the behaviors displayed by these dolphins indicated flexible, complex, sequence rule learning. The “language” also allowed testing of a variety of concepts: presence vs. absence of objects, identification of body parts, memory for action events such as repetitions after delays of behaviors, combinations of behaviors, and actions on a specific object in the presence of many other objects (Mercado and Delong, 2010).

Dolphins have also learned sequences in other contexts. They can recognize relative number magnitudes in ordered sequences and novel melody sequences as ascending or descending (review in Pack, 2015). They can recognize specific rhythms, transfer them across frequency and tempo shifts, and produce them (Harley et al., 2005). In a task in which they were originally trained to produce rhythms using a pneumatic switch, they spontaneously transferred the rhythms to vocalizations. The transfer suggests abstract representation of the rhythm and/or ability to copy a tonal rhythm.

Dolphins interpret and produce referential pointing gestures when engaging with another species. They follow referential human points (i.e., pointing to an object; Herman, et al., 1999), as well as using pointing gestures themselves to direct humans (Xitco et al., 2001a,b). In these studies, dolphins pointed with their rostrums at fish in jars placed at various locations in a captive habitat in the presence of humans but not in their absence. The dolphins would also engage in joint attention behaviors by turning toward human swimmers and pointing back at the jars. The humans responded by opening the jars.

The flexible referential quality of dolphin cognition, in contrast to perception of the simple, physical stimulus character of objects, is illustrated in cross-modal experiments. Harley et al. (1996, 2003) and Pack and colleagues (Pack and Herman, 1995; Herman et al., 1998) trained dolphins to identify objects in one modality (vision) and then identify them in a different one (echolocation) and vice versa using a matching-to-sample format. For example, a dolphin wearing eyecups to block vision investigated an object echoically but then successfully matched that object to an identical alternative presented visually in air where dolphin echolocation does not work. Since the visual and echoic (hearing) experiences are obviously physically different, the dolphin had to represent the stimuli in such a way as to allow recognition in either modality. Dolphins clearly have a plastic hierarchical object representation system that includes attributes gleaned through multiple, high-resolution sensory modalities.

Flexibility is also required to identify objects using echolocation alone in that the echoes from different aspects of a single object can vary more than those between different objects (review in Harley and DeLong, 2008). For objects that vary only in size, like different-sized disks, dolphins can use differences in amplitude to discriminate among the disks. For objects that vary only in material, like an aluminum vs. a stainless-steel cylinder, dolphins may use different pitches to tell the objects apart. Once objects get more complex and vary across many features simultaneously, it is more difficult to know how the dolphins manage recognition tasks because echoes from different attributes interact, but they can do it. This same quality of the elasticity of dolphin cognition is evident in the interpretation of shared echoes. Xitco and Roitblat (1996), using a three-choice delayed matching-to-sample task, demonstrated that a dolphin who had only heard the echoes returning from a sample object to its neighbor could choose the identical alternative at above-chance levels. The import of this ability to share information directly is that it may allow a group of dolphins to act as a sensory integration unit (Norris and Johnson, 1994), surpassing the experience of any individual.

Anecdotal evidence, corroborated by experimental work, suggests cetacean planning abilities. For example, killer whale foraging and dolphin sponge fishing discussed earlier indicate some preparation. Two experimental studies provided more easily verifiable evidence of planning (review in Kuczaj and Walker, 2006). In the first experiment, dolphins learned a task in which four weights placed in a device within a given timespan released a fish. Dolphins learned the task by observing a human placing the weights one at a time and then executed the task in the same way when weights were close to the device. When weights were further away, the dolphins switched strategies to carry multiple weights to the device, a more efficient approach suggesting planning. In the second experiment, three separate devices released a fish when one weight was inserted. Two of the three devices allowed the weight to pass through, so it could be reused. The third did not release the weight, preventing the dolphin from getting fish from the other devices. The best strategy, therefore, was to select this device last, which the dolphins learned to do.

### Pinnipeds

Although behavioral and cognitive studies of pinnipeds have featured small sample sizes, and have focused predominantly on California sea lions, impressive results have been obtained in the realm of language learning, memory, concept formation, and rhythmic capability. As with the cetaceans, the number of apparently unique and rare abilities observed in pinnipeds is striking given how few studies with how few subjects there have been.

While studies examining human-like language learning in animals have mostly featured apes and cetaceans, there were a series of studies in the 80s and 90s with sea lions. As reviewed in Schusterman and Gisiner (1997), several sea lions, having learned to respond to gestures indicating objects (e.g., cones and balls), descriptors (e.g., large and white), and actions (e.g., fetch and bring to), responded appropriately to novel combinations of those gestures (e.g., bring the small black ball to the large white cone). This suggests something akin to receptive syntax, which has been shown in very few non-human species.

Further studies at the same laboratory probed the ability of sea lions to group arbitrary stimuli into concept classes and then to use logical reasoning to add new stimuli into one class or another with one-trial learning. For example, having learned that A, B, and C go together, and 1, 2, and 3 go together, sea lions were able to add D to the correct class following one exposure. In other words, if D goes with A, B and C go with A, D must also go with B and C. Further, because A, B, and C do not go with 1, 2, and 3, if D goes with A then D does not go with 1, 2, and 3. This represents a type of transitive inference rarely demonstrated in non-human animals. Impressively, sea lions have demonstrated robust long-term (10+ years) memory not just for the stimuli involved in these experiments but for their logical relations to each other (Schusterman et al., 2002).

Sea lions are also unique among non-human animals for having shown the ability to move in time to a musical rhythm and then to generalize it to novel stimuli and tempi in transfer tests (Cook et al., 2013). This capacity was previously believed unique to humans until demonstrated in some parrot-type birds (Patel et al., 2009), leading to the theory that brain circuits involved in complex vocal production learning were necessary to learn to match movement timing to complex auditory rhythms as in human dance. Sea lions, who show very limited vocal learning, challenge this theory. It remains to be seen how widely this faculty is distributed. It may be that, as a motivated animal with strong motor control, sea lions have an easier time demonstrating certain complex sensory-motor behaviors than many other species (Wilson and Cook, 2016).

A patchwork of studies over the last 15 years has probed a number of “higher” cognitive abilities in sea lions related to self-control, working memory, and mental manipulation of representation. While more work is needed, sea lions have shown strong inhibition of pre-potent motor responses, besting primates in their capacity to inhibit selection of a lesser reward for later receipt of a greater (Genty and Roeder, 2006; though see Beran and Hopkins, 2018). Sea lions can also mentally rotate shapes in matching tasks. While orientation-invariant matching is not rare in tested animals, sea lions are unusual in that their response times scale with the degree of mismatch between the stimuli and their comparisons (Mauck and Dehnhardt, 1997; Stich et al., 2003). One explanation is that, as humans are believed to do, they are actually rotating a mental representation in working memory. They have also demonstrated the ability to locate objects based solely on mirrored visual representations (Hill et al., 2015). In addition, they show a capability to follow ostensive pointing gestures with high success, potentially relating to an ability to decouple a local visual stimulus from its immediate surroundings (Scheumann and Call, 2004). Finally, South American sea lions have been shown to have primate-like capabilities for discriminating stimuli based on numerosity, a skill generally believed reliant on some degree of working memory function (Abramson et al., 2011). Each of these abilities could be considered to be related to “executive function,” a general set of neurobehavioral processes relying on prefrontal and parietal associative “control” regions in humans and primates.

### Sirenians

Sirenians, primarily manatees, display basic discrimination learning abilities in studies of sensory detection and discrimination thresholds in the tactile, auditory, and visual domains (review in Bauer and Reep, 2017). There has been no formal research on long-term memory in manatees. Anecdotal evidence from Florida manatees in the wild is suggestive. Reep and Bonde (2006) report that manatees recall the location of freshwater hoses between seasons. In a captive situation, two manatees remembered an active touch discrimination of textures procedure with 100% performance accuracy after 14 and 29 months, respectively (Bauer et al., 2012). Cognitive investigations limited primarily to response shaping and discrimination learning does not provide an adequate basis for conclusions characterizing sirenian intelligence.

Cetaceans and pinnipeds display a wide range of cognitive abilities. Perhaps the issue is not so much their ability at any one of the procedures on which they have been tested, many of which have been displayed by diverse other species, but in the range of abilities demonstrated. Of significance for intelligence is the complexity of representation and the transfer of complex skill sets across contexts highlighting flexible intelligence, e.g., in cross-modal tasks. It is also striking that a relatively small number of marine mammal subjects has demonstrated such an expansive list of abilities.

## The Brain

To the extent that conceptions of intelligence rely on association, sensory processing, representation, and manipulation of information, intelligence can be understood to be a general feature of the nervous system, or, at least, a general potential for the nervous system to produce certain outcomes in different environmental contexts. Human neuroscience, bolstered by functional neuroimaging technology, has done much to unravel the neurobiological mechanisms undergirding human cognition. We now have a strong understanding of which brain regions represent sensory information, which brain regions code motor behavior, and the relation between these sets of regions that allow us to respond to our environment (Power et al., 2011). These primary brain regions, directly connected to body sensors and effectors, are evolutionarily conserved and provide the foundation for the brain’s higher processes. We further have delved into how non-primary “association” regions in the brain, with no direct connection to body sensors or effectors, work to regulate, control, and manipulate primary brain regions to support complex cognition (Goldman-Rakic, 1988). The human brain is composed of parallel hierarchies of motor and sensory processing (Fuster, 1997). The primary motor and sensory regions are cortical brain regions directly connected to body sensors and effectors. These areas share information with, and are regulated by, secondary cortical brain regions with no direct connections to body effectors, the premotor cortex and unimodal association areas, respectively. These secondary regions in turn share information with and are regulated by tertiary regions that influence the secondary regions, and, typically, through those secondary regions, the primary regions connected to the body. These are the prefrontal cortex in the motor hierarchy and the polymodal association cortex in the sensory hierarchy, and they can be thought of as sitting atop the neural hierarchy, exerting disproportionate control over the other brain regions. The influence of prefrontal and polymodal association areas is strongly correlated with “higher” cognitive function in humans, allowing the formation, maintenance, storage, and manipulation of complex representations (Yeo et al., 2015).

In well understood examples drawn from human neuroscience, primary sensory regions can be recruited by the hippocampus and prefrontal cortex to support experiential memory (Preston and Eichenbaum, 2013). Motor regions can be inhibited by frontal control regions to stop immediate response to stimuli, opening up time for slower, more deliberate responses and planning (Ridderinkhof et al., 2004). Subcortical regions processing reward can be activated in concert with memory supporting and motor control regions to support complex learning and planning based on prior and simulated outcomes (Pasupathy and Miller, 2005). In these and essentially all other circumstances of higher cognition in humans, our current neuroscientific understanding relies on *connectivity* (see Rubinov and Sporns, 2010). Brain regions influence each other through connections, the patterns of these connections are a map of potential interactions and thus potential neurobehavioral outcomes, and the dynamic interaction and plasticity of these regions and their connections support complex and changing behavior across a range of situations.

Comparative neuroscience now also increasingly operates on a connectionist framework (Mars et al., 2016, 2018), and studies of rodents and primates seek to find the similarities and differences related to network connectivity in humans, in order to better understand the functional relevance of these connections and how they influence behavior, both typical and atypical as in disease states. Marine mammals have long been of interest to comparative neuroscientists for a number of reasons, but the bulk of interest has been driven by the grossest features of their neurobiology. First, size – marine mammal brains are large in comparison to those of terrestrial animals, both in absolute terms, but also, for some species such as dolphins, in relation to body size (Marino, 1998). Second, gyrification – the pattern of folds (including bumps, or gyri, and grooves, or sulci). Pinnipeds and cetaceans have remarkably folded brains in comparison to terrestrial mammals, while sirenians have remarkably smooth (lissencephalic) brains (Reidenberg, 2007). Most research on marine mammal brains to date has addressed two general features – size and wrinkliness, and we will briefly review that literature below. A number of researchers have suggested that the large size of cetacean brains, when viewed from the perspective of their often impressive behavior in the laboratory, is a clear indicator of extreme intelligence (Marino et al., 2007). Fewer hypotheses have been advanced regarding the functional relevance of gyrification, and recent research suggests gyrification is predominantly a product of brain size and neuron proliferation early in development (Mota and Herculano-Houzel, 2015).

It must be emphasized that, from the perspective of modern neuroscience, while size does matter, this is mostly as it relates to the number of neuron units (Herculano-Houzel, 2009). Neurons are the general information processing unit of the nervous system (Shepherd, 2015). More neurons mean the potential for more processing power. It turns out that the correlation between brain size and neuron number across species is, while present, fairly variable (Herculano-Houzel et al., 2014). Further, the number of neurons, while important, is no more (and possibly less) important than the patterns of connections between those neurons and the regions they compose (e.g., in humans, Emerson and Cantlon, 2012; Xiang et al., 2012; Xiao et al., 2018). Here, research into marine mammal neurobiology is still in its infancy. We discuss preliminary efforts to characterize marine mammal brains from the perspective of functional processing, and suggest some potentially fruitful and achievable future directions that will better enable us to understand in which ways marine mammal nervous systems are like and unlike those of their terrestrial relatives. In line with the Jamesian principle that cognition and intelligence are reliant on features of neurobiology, this may help support a framework for assessing the general intelligence of these species.

### Marine Mammal Brain Size

On the topic of sheer size, marine mammals are notable for featuring the species with earth’s largest brain, the sperm whale. Weighing in at up to 8 kg (Povinelli et al., 2014), it dwarfs the human’s 1.3 kg brain. In addition, marine mammals include two of the four clades in the “over 700 g club” (Manger et al., 2013) with numerous whales, and four separate pinniped species besting this brain weight. Generally speaking, animals with bigger bodies have bigger brains, so it is perhaps not surprising that, freed from the constraints of gravity in a terrestrial environment, marine mammals evolved bigger bodies, and, thus, bigger brains. The terrestrial members of the club are apes and elephants. Apes may seem an exception compared to other club members in the relatively small size of their bodies. Indeed, apes are particularly notable for their “encephalization quotient (EQ),” a measure comparing brain-to-body-size ratio against the typical cross-species trend (Jerison, 1977). Some have suggested that EQ can serve as a predictor of a species’ intelligence (Jerison, 1985). The human EQ is up to seven times what would be expected for their body size. Some of the toothed whales, particularly dolphin species, also have very high EQs, up to four times what would be expected based on body size (Marino et al., 2004). Despite their large brains, baleen whales and pinnipeds suffer on EQ measures, tending to fall close to the average brain size predicted by body size (Worthy and Hickie, 1986). Just as an unusually large brain increases EQ, so does an unusually large body decrease EQ. This may be the case with manatees. The attributions for sirenian intelligence based on brain size may suffer from the simplistic view that the small relative brain/body ratio of manatees and dugongs (Jerison, 1973) implies a dim intellect. O'Shea and Reep (1990) argue that this is a misrepresentation that does not take into consideration ecological (herbivory) and physiological (heat conservation) pressures driving large body size. That is, sirenians do not have excessively small brains, rather they have disproportionately large bodies.

Reliance on EQ as a predictor of intelligence has faded, with some evidence suggesting that, within related clades of animals, overall brain size is a stronger predictor of cognitive capability than EQ (Deaner et al., 2007). This returns us to the importance of overall brain size, but, again, the size of the brain is most notable as it predicts neuron count (processing power). Just because a brain has evolved to be bigger does not mean it will have more neurons. In fact, animals with bigger bodies tend to have less dense “neuronal packing.” For example, some bird species have as many neurons packed into their forebrains (analogous to mammalian cortex) as some primate species (Olkowicz et al., 2016). A gray parrot’s brain weighs no more than 20 g, while a lion’s brain is over 10 times that size. But the gray parrot has twice the cortical neuron count of a lion. These birds have a much higher measure of processing power per unit of brain volume than do mammals. Obtaining neuron counts used to be prohibitively time consuming, but newer methods allow much more rapid counting (von Bartheld et al., 2016). Notably, the current record holder for total number of cortical neurons is the killer whale, with over 40 billion cortical neurons (Ridgway et al., 2019). The pilot whale is a close second, with 32 billion (Mortensen et al., 2014), twice what humans average at 16 billion (Herculano-Houzel et al., 2015). Most other cetaceans measured, including dolphins, have 10–12 billion cortical neurons, slightly more than the 7–10 billion found in non-human ape species (Herculano-Houzel, 2019). Pinnipeds have fewer, although still relatively high numbers compared to terrestrial mammals. Elephant seals and walruses, the biggest pinnipeds, have in the range of 4 billion cortical neurons. Contrast this to a horse, with approximately 1 billion (Haug, 1987), or a dog, with 500 million (Jardim-Messeder et al., 2017). These numbers help contextualize prior debates about the relevance of cetacean brain size. While a controversial hypothesis (Manger, 2006) has suggested that cetacean brain size is largely due to thermoregulation, the high neuronal cell counts better match other theories emphasizing cetacean cognitive capability (Marino et al., 2007) – not because brains are bigger, but because we now know they likely have more processing power.

However, it is not just the number of neurons that matters, it is where they are, and how they are connected. More work is needed to obtain neuron counts from specific structures in marine mammal brains. For example, much has been made of the small gross volume of the dolphin hippocampus, a region involved in explicit memory processes in mammals (Oelschläger, 2008). Cell counts of different regions will provide a better indicator of those regions’ importance and functional capabilities. It does appear that toothed whales have densely packed brains with many cortical neurons. But what regions are those neurons in? Great expansion of primary processing regions, as seen in cortical enlargement of motor control systems in human hand cortex and visual cortex in primates (Krubitzer, 2007; Kaas, 2008), can increase brain size, relative brain size, and total cell count but may have quite different relevance for assessing global intelligence than parallel increases in association cortices and other brain regions “higher” in the neural processing hierarchy.

As noted, it is this pattern of connections that allows a region to engage in a specific function. Indeed, regional definition depends predominantly on connection profile. The cortical region receiving the bulk of direct projections from auditory receiving structures will be the primary auditory cortex, regardless of where it is in the brain. Contemporary neuroscientists believe these patterns of connection represent the possibility space for a nervous system. Everything that a nervous system can do, including information processing of the sort we tend to consider “cognitive” or intelligent (memory, decision making, self-control, and learning) relies on communication across specific connections between different brain and body regions.

Human neuroscience has placed particular emphasis on corticocortical connections – communication pathways between different cortical areas that support dynamic and flexible information processing. Here, as in the literature on size, whale and dolphin brains have received the most attention among marine mammals. Early histological work characterized the whale cortex as “primitive,” meaning similar in some ways to non-placental mammals like monotremes and marsupials, taken to be emblematic of early mammal neurobiology (Morgane et al., 1985). Unlike most extant terrestrial mammals, whale cortex has five instead of six discrete cell layers. Ancestral mammals were believed to have five, while the vast majority of extant species have six, suggesting whales lost a layer somewhere in their evolutionary history after returning to the water (Barbas and Rempel-Clower, 1997).

The missing layer, cortical layer 4, is essential for connecting distributed cortical regions in terrestrial mammals (Dantzker and Callaway, 2000), and its absence, in addition to the sparse cross-hemispheric connections in cetaceans, has been taken as evidence for generally low corticocortical connectivity in the whales and dolphins. Importantly, cross-hemispheric connectivity may be reduced in part to allow for unihemispheric sleep (Tarpley and Ridgway, 1994). More recent histological examination of whale and dolphin cortex has indicated unusual patterns of dense local connectivity (Hof et al., 2005). In addition, whales do have some features associated with complex long-distance brain connectivity, such as giant “spindle” neurons also found in elephants and primates (Butti et al., 2013; Raghanti et al., 2015). Hof et al. have suggested that whale brains are not under-connected but, rather, *differently* connected. What the cognitive ramifications of this altered connectivity might be remains to be determined.

Decoding and interpreting the patterns of connectivity in whale brains will require identification of functional processing regions – as stated, it is the connection between these regions that forms the basis of brain architecture. Traditionally, neuroscientists have conducted careful cell staining studies (histology) to characterize different neural populations associated with different processing regions. One of the potential mysteries of cetacean neurobiology is the apparent lack of differentiation in cortical cell type across regions (Morgane et al., 1980; although see again Hof et al., 2005), frustrating attempts to localize functional processing regions by cell type. There have been fewer attempts to conduct these types of analyses in pinnipeds, but recent studies have successfully delineated somatosensory and visual cortex in pinniped species (Sawyer et al., 2016; Turner et al., 2017). Pinniped somatosensory cortex is large, well developed and has a high proportion of cells involved in receiving and processing touch signals from vibrissae (whiskers). When possible, these types of histological analyses can speak to the characteristics of primary sensory and motor regions and may help determine the volume and type of information processing these regions can afford.

Functional brain regions can also be identified *via* tracing studies. Historically, tracing has been conducted with chemical agents that are injected directly into a brain region, and then transported (forward or backwards along axonal connections, depending on the agent used) to connected regions (Oztas, 2003). These injections are administered to a live animal that is then killed, the brain removed and sectioned to find transport sites. Such work is no longer conducted in marine mammal species for ethical and regulatory reasons, but early work with cetaceans did seek to identify cortical processing regions for auditory information (obviously of interest given complex vocal communication and echolocation in many cetacean species). These studies indicated that primary auditory cortex was in the dorsal posterior portion of dolphin cortex, in or adjacent to the cortical regions where primary visual processing typically occurs in mammal brains (Sokolov et al., 1972; Popov et al., 1986). These studies have typically indicated reduced or absent association cortex separating these primary processing regions, which would suggest a very unusual overall pattern of brain organization, potentially relevant to how cetaceans process and integrate echoic and visual signals.

While transport tracing is no longer plausible for use in marine mammals, a set of non-invasive neuroimaging techniques can provide similar information about connection between different brain regions. Diffusion tensor imaging (DTI) is an application of magnetic resonance imaging (MRI) technology, relying on determination of direction and magnitude of water movement in the brain (Le Bihan et al., 2001). Water moves most reliably and easily along large axons, which form the primary pathway for neural communication in the brain. Thus, DTI can provide a map of the structural connections in the brain. These images can be acquired from live animals (although this requires anesthesia, which comes with risks, particularly in some of the marine mammals with non-obligate breath control). They can also be obtained from dead brains. If the images are acquired soon after the brain is removed (e.g., following planned euthanasia or a stranding death), the images can be as good or better than those obtained in live brains (Seehaus et al., 2015). Recent applications of post-mortem DTI have yielded tantalizing new information about the connectivity of dolphin auditory systems. Berns et al. (2015) traced connections from the inferior colliculus in the dolphin midbrain, the primary midbrain waystation of ascending auditory information, and found strong projections to the superior temporal lobe, significantly less dorsal than transport tracing studies have indicated the location of primary auditory cortex to be, and in line with primary auditory projections in terrestrial mammals. In addition, researchers have begun to map auditory-motor pathways in dolphin brains that may be analogous to the arcuate connections supporting vocal learning in humans (Wright et al., 2018).

These techniques are now being applied to pinniped brains in an effort to map out auditory-motor connections (relevant to ongoing debate over vocal learning capabilities in pinniped species). They have also been used to map specific neurological damage in wild sea lions exposed to algal toxins (Cook et al., 2018).

Mapping connection patterns in marine mammal brains will help us understand the functional architecture of these brains and determine to what extent it differs from that of terrestrial mammals. For example, if we can delineate the dolphin auditory cortex based on patterns of connectivity with lower brain regions, we can begin to determine to what extent auditory expansion accounts for overall brain expansion. More importantly, we can assess whether the patterns of connections with auditory regions support the complex, multi-region hierarchical processing we associate with higher cognition in humans. For example, the apparent lack of dolphin frontal cortex (cortical regions anterior to motor regions) has been commented on frequently in the literature. Berns et al. (2015) used projections from basal ganglia regions to map out brain regions functionally analogous to prefrontal regions in humans and found that they largely paralleled those observed in other species, although the gross location of regions was somewhat more lateral. Anecdotal assessments of corticocortical connectivity in pinnipeds (high) and manatees (low) may also lead to quantifications related to the capabilities of those species and can be used to assess potential functional relevance of gyrification patterns. The density and patterns of these connections in marine mammals, and how they compare to those in humans and other terrestrial mammals, will provide a biological framework for considering behavioral measures associated with intelligence and flexible cognition. In addition, by collecting neurobiological data from more individuals, we can begin to assess inter-individual variability in brain organization, which should bear directly on individual differences in cognition and behavior.

## Discussion

We suggest that the transition from a terrestrial to a marine environment encouraged an emphasis on high-resolution auditory and tactile senses, while reducing the importance of visual and chemical modalities. The high resolution of hearing and touch promoted stimulus discrimination capabilities. Furthermore, the high speed of sound in water required faster information processing as reflected in high temporal resolution, rapid sound integration, and good sound localization. The absence of solid physical structures for hiding from predators and prey in three-dimensional aquatic space facilitated the development of social grouping for many marine mammals for defense and foraging. Group living, in turn, fostered the development of an array of social learning skills, particularly mimetic behaviors, unsurpassed by other mammals other than humans. Marine mammals also demonstrate a wide array of other flexible cognitive capacities, perhaps surprising and notable given the relatively small number of animals tested a small number of times. What does this tell us about marine mammal intelligence?

The diversity of definitions or characterizations of “intelligence” makes this a difficult question to answer. Macphail’s (1982) characterization, which limits animal intelligence to associative processes, simply does not capture the way “intelligence” is used by the lay public or professional researchers of human intelligence. Mackintosh (1998) notes that the types of associative processes Macphail describes are largely subsumed under implicit learning in human research and are not assessed on intelligence tests. That is, human intelligence research segregates intelligence from the very characteristics that Macphail characterizes as animal intelligence, a difficult position to reconcile with an evolutionary perspective. In many ways, “intelligence” seems to be a folk psychology term that maps poorly on natural psychological and biological processes, and therefore, lends itself to a wide range of often-inconsistent interpretations.

In the absence of a generally agreed upon theory of intelligence, we ought to ask what approaches lead to fruitful lines of inquiry, inquiries that might lead to theory development. Researchers investigating questions of marine mammal behavior have generally found it productive to address brain-behavior relationships, ecological adaptations, and comparisons among species. Early efforts to study cetacean cognition engaged in an overly simplistic attempt to confirm a speculative hypothesis that dolphins and whales, because of their large brains, must be highly intelligent (Lilly, 1967). This approach was given some credibility by the fact that some species of cetaceans had large brain-to-body relationships (Jerison, 1973) and that the largest of the toothed whales, the sperm whale, had the largest brain in terms of absolute size of all animals (Oelschlager and Kemp, 1999; Povinelli et al., 2014). While it is true that, within humans, larger brains have been correlated with higher measures on intelligence tests (Lee et al., 2019), this may be due to the correlation between brain size and neuron number within a species. Across species, the correlation between number of neurons and brain size is only moderate, as “neuronal packing” density can differ greatly. Adherence to the big brain-high intelligence hypothesis has yielded to more fine-tuned approaches emphasizing structure, organization, and function of brains. Cell counts suggest that some marine mammal brains are unique in terms of the sheer number of neurons they contain, e.g., killer whales and pilot whales have more than twice the number of cortical neurons than humans do. We have begun to explore overall patterns of histology and connectivity to identify different brain regions and map the patterns of connections between them with the goal of determining functionality. These data are getting easier to acquire and manipulate and could lead to specific hypotheses concerning what types of information processing are strengths of these animals. Hof et al. (2005) have suggested that in addition to large brains, cetaceans have unique patterns of cortical connectivity that, although different in structure from that of terrestrial mammals, may allow for formation of complex associations and manipulation of complex representations. While connectivity has been linked with variability in intelligence in humans (Song et al., 2008), it is important to note that at the most simple level, brain connections allow associations between different regions. Thus, differences in connectivity alone are not enough to refute an account such as Macphail’s, where conserved associative learning is broadly shared across species. That said, different connection patterns may allow vastly different behavioral and cognitive outcomes, so must be considered. Although considerations of brain-behavior relationships in marine mammals are still emerging, we nevertheless think consideration of brain structure and function as opposed to mere size is the appropriate approach for better understanding cognitive/behavioral attributes.

The benefits of an ecological approach to intelligence can be seen most clearly in the sensory realm, where marine mammals demonstrate acute sensitivity and discrimination of sound and tactile stimuli. The capacities of the visual senses are more varied among marine mammal species, although for many their acuity is also quite good. The resolving capabilities of marine mammals provide a rich *Umwelt* in the acoustic and tactile realms, one that implies a detailed perceptual texture to their lives. The ecology of these animals drove these sensory changes to allow them to marshal their cognitive power to respond flexibly to their new surroundings. The quantitative precision with which sensory sensitivity, discrimination, and identification can be measured also facilitates comparisons to other species. The high correlation between general sensory discrimination and fluid intelligence (and perhaps working memory) in humans also suggests an avenue for further intelligence research in animals.

In addition to sensory adaptations, the transition to the ocean also facilitated social adaptations. For many species, social grouping fostered the sensory integration and behavioral coordination among members necessary for successful hunting, defense, and other activities (Norris and Johnson, 1994) in the absence of much of the physical scaffolding used by terrestrial animals. Group coordination placed a premium on social learning among marine mammals, and it is in social mimicry where a clear difference is found between cetaceans and terrestrial mammals. They are the only mammals other than humans reported to demonstrate vocal and behavioral copying behavior beyond mere rudiments. This copying behavior is strikingly flexible, characterized by learning novel skills, demonstrating both accurate mimicry of physical movement and emulation of end goals, mimicking the behavior of other species, mimicking computer-generated sounds, and copying behaviors of other species, even humans in air. Evidence for social learning in the wild, although not as tightly controlled as in the laboratory, indicates that the abstract learning situations tested in the laboratory have practical generality to the natural environment. For example, vocal mimicry is reported from observations and experiments with bottlenose dolphins in the laboratory (Richards et al., 1984; Richards, 1986) and in the wild (Janik et al., 2006). Acquisition of novel motor behavior is also reported in the laboratory (e.g., Xitco, 1988; Xitco et al., 1998) and in the wild (Wild et al., 2020). Social behavior and vocal imitation provide another rich area for comparative work on intelligence.

While some of the cognitive skills tested in cetaceans and pinnipeds are found in other species, the breadth in marine mammals is marked. For example, although animals with few neurons in their nervous systems, like honeybees with a million neurons, can do delayed matching-to-sample tasks, marine mammals’ neuronal tool kit (supported by perhaps a million times that number in killer whales, for example) seems to be expanded. An approach that considers intelligence to be multifaceted considers a wide range of test performances; intelligence might be assessed over procedures testing myriad capacities, for example, perceptual resolution, short and long term memory capacity, imitation, problem solving, and the many other attributes suggested by Bullock (1986). Both cetaceans and pinnipeds have demonstrated successful performance on a broad array of tasks. The relative range compared to other species remains to be evaluated.

Several other factors could be incorporated into a model of intelligence: (1) Analysis of cognitive representation in addition to measurement of stimulus features can provide insight to the way animals make connections. For example, showing that a dolphin can identify visually an object that has previously only been identified through audition and vice-versa indicates a representation independent of modality. (2) The intelligence of a species might be indicated by its ability to learn from experience. In this case, we are talking about more than just a learning set type of experiment but rather the changes that occur over days, weeks, and years showing learning built on previous experiences. Many researchers report anecdotally that marine mammals who engage in years and decades of cognitive work improve in their ability to learn new test procedures over time. Such long-term growth and change are fundamental to our understanding of human intelligence, and the long developmental course of many marine mammals suggests extended neural and behavioral plasticity, as seen in humans. There is now some evidence that behavioral plasticity is, indeed, adaptive (Ducatez et al., 2020), allowing some species to better adjust to and survive in rapidly changing environments. If flexibility and the knowledge attainment it supports are adaptive, then they are subject to evolutionary pressures and will necessarily vary across species. It is possible that comparative psychologists have unintentionally gone out of their way to ignore these factors by focusing study on naive animals placed in impoverished contexts; this method might squelch our ability to find differences across species and between individuals. (3) Anatomical and physiological techniques can greatly enhance the collection efficiency of experimental data. One of the big problems of marine mammal behavioral research is the length of time it takes to collect data with all its attendant costs, small sample sizes, and limits on questions to be asked. For example, a visual acuity test or audiogram for a naïve animal might take a year. Alternatively, good estimates of visual acuity can be determined from measures of retinal ganglion cell density and axial length of the eye (Mass and Supin, 1989), measurements that can be quickly made post-mortem. Good audiogram approximations can be made through evoked potential techniques in less than an hour (Finneran and Houser, 2006). As neural function and organization measurement improves, we may be able to explore valid cognitive characteristics through widely available anatomical techniques like post-mortem DTI. (4) Differences in individual intelligence are a major focus of human intelligence testing, but we do not usually consider this quality in comparing intelligence among species. This is certainly something that animal trainers encounter, when they find major differences in trainability among subjects, although it may not be something that is formally assessed and reported. Variability in intelligence among individuals might reflect the cognitive flexibility of a species better than a static measure of average performance.

Just because comparative psychologists have yet to successfully characterize and delineate all the processes and situations that govern animal thought and behavior does not mean that there are not significant differences in how animals gather information in the world, process it, and act on it across multiple contexts. As indicated here, there are numerous comparisons we could make that might be more fruitful for delineating differences in intelligence than the foundational processes targeted by Macphail. Clearly, these foundational processes exist, but they are recruited differentially across species as their ecologies drive shifts in other systems (e.g., sensory-motor ones) bringing new information to their *Umwelten* and expanding fundamental areas of cognition (e.g., through requiring much faster temporal processing to deal with sound in the water). Marine mammal species transitioned, over the course of evolutionary history, between markedly different ecological settings, and continue to transition between these settings on a daily basis. These transitions may have promoted neural, sensory, and cognitive flexibility reflected in their behavior in the wild and in the laboratory. As long-lived animals who perform well in experimental settings, they are excellently situated to provide insight into the link between ecological and cognitive flexibility and how this may bear on a comparative understanding of intelligence.

## Data Availability Statement

The original contributions presented in the study are included in the article/Supplementary Material, further inquiries can be directed to the corresponding author.

## Author Contributions

The work was a collaborative effort among HH, PC, and GB. All parts of the manuscript reflect a group effort with the exception of The Brain, which was written primarily by PC. All authors contributed to the article and approved the submitted version.

### Conflict of Interest

HH participates in scientific and welfare collaborations with Walt Disney World’s Animals, Science, and Education. The Seas, Epcot®, Walt Disney World® Resorts was not involved in the study design, collection, analysis, interpretation of data, the writing of this article or the decision to submit it for publication.The remaining authors declare that the research was conducted in the absence of any commercial conflicts of interest.
